# A Structured Computational Roadmap for Lipidomics in R: Reproducible Workflows from Raw Data to Functional Insight

**DOI:** 10.3390/metabo16050288

**Published:** 2026-04-22

**Authors:** Maria-Christina P. Papatheodorou, Panagiotis Vlamos, Marios G. Krokidis

**Affiliations:** Bioinformatics and Human Electrophysiology Laboratory, Department of Informatics, Ionian University, 49100 Corfu, Greece; papatheodorou@ionio.gr (M.-C.P.P.); vlamos@ionio.gr (P.V.)

**Keywords:** lipidomics, R libraries, lipid ontology, functional analysis, data processing, multi-omics integration

## Abstract

Lipidomics has emerged as a transformative discipline in biomedical research, providing high-resolution insights into metabolic signaling and disease pathophysiology. The R programming language provides a widely adopted framework for extensible analysis of complex lipidomic datasets due to its robust biostatistical infrastructure. Herein, we present a comprehensive roadmap for lipidomics in R, structured around a standardized analytical lifecycle: from raw data acquisition and preprocessing to structural annotation, statistical modeling and functional interpretation. We critically contextualize and integrate a curated suite of widely adopted R packages (version 4.3.0), including xcms and MSnbase for feature extraction, LipidMS 3.0 for fragmentation-based identification, and lipidr for quality control and normalization. Furthermore, we demonstrate how advanced tools such as mixOmics and clusterProfiler can be integrated to bridge the gap between differential lipid abundance and systems-level biological insights. Particular emphasis is placed on reproducibility, nomenclature standardization and the emerging role of machine learning in biomarker discovery. By synthesizing these resources into a coherent pipeline, this guide provides a structured reference for researchers. Further discussion addresses methodological pitfalls, statistical assumptions and reproducibility constraints that frequently compromise lipidomics studies. Ultimately, this structured approach facilitates systematic tool selection, accelerating the translation of complex lipidomic signatures into reproducible and clinically meaningful discoveries.

## 1. Introduction

Lipidomics has emerged as a rapidly advancing field of biomedical and translational research, offering high-resolution insights into lipid metabolism, cellular signaling, and the molecular mechanisms underlying complex diseases. Systematic changes in lipid composition are fundamentally linked to cardiometabolic disorders, neurodegeneration [1], cancer progression and immune dysregulation, positioning lipidomics as a critical source of clinical biomarkers and therapeutic targets [2,3]. However, despite its diagnostic potential, the field continues to face significant hurdles arising from a fragmented and inconsistent computational landscape [4,5].

As highlighted by Ni et al. (2022) [6], the proliferation of informatics tools has created a complex environment where selecting the appropriate software for specific research applications remains a formidable challenge [7]. Furthermore, recent trends in computational metabolomics (2021–2025) underscore a critical shift toward high-performance, automated platforms, yet the integration of these disparate tools into a cohesive workflow remains incomplete [8]. To address this ambiguity, this article is structured as a narrative review that functions as a decision-making roadmap for R-based lipidomics. Unlike a simple catalog of tools, we propose a curated workflow based on a transparent selection strategy. Packages discussed in this roadmap were identified through the Comprehensive R Archive Network (CRAN) and Bioconductor repositories and selected based on four objective criteria: (i) methodological rigor and peer-reviewed validation, (ii) active maintenance (defined by updates within the last 24 months), (iii) high interoperability with standardized S4 data structures (e.g., SummarizedExperiment), and (iv) community adoption, prioritizing tools with extensive documentation and stable user bases.

As lipidomics applications expand, the demand for robust computational pipelines capable of handling high-dimensional and heterogeneous datasets has grown substantially. The R programming language has emerged as the most widely adopted platform for metabolomics and lipidomics, largely due to its mature Bioconductor infrastructure and its long-standing role in biostatistical computing [9]. Originally developed as a statistical environment in the early 1990s, R has evolved into a comprehensive framework supporting the entire analytical lifecycle—ranging from raw mass spectrometry (MS) data ingestion and preprocessing to lipid annotation, statistical modeling, and systems-level biological interpretation [9,10].

An increasing number of R packages now provide specialized functionality tailored to the unique requirements of lipidomic data. Established tools such as xcms [10] and MSnbase [11] support robust peak detection and raw data management, while LipidMS 3.0 [12] enables fragmentation-driven lipid annotation. Downstream, lipidr [13] offers an integrated environment for quality control, normalization, and differential analysis, while high-dimensional visualization and biological interpretation are facilitated by packages such as ComplexHeatmap [14] and clusterProfiler [15]. The landscape of these informatics tools has been recently synthesized in several comprehensive reviews; for instance, Ni et al. (2022) [6] established a foundational guide for software selection based on lipid structural resolution and mass spectrometry (MS) platform compatibility [7]. More recently, Nafie et al., summarized the broader computational trends in metabolomics, emphasizing the shift toward high-throughput automated discovery and cloud-based infrastructures [8]. The major strength of the R ecosystem lies in the interoperability of these tools, enabling seamless transitions across the various analytical stages as well as on nucleic acids and protein analyses [16]. The efficacy of these tools, however, depends on their strategic integration. Despite the rapid expansion of R-based lipidomics tools, the absence of a unified decision framework often results in inconsistent analytical strategies, suboptimal statistical modeling, and limited cross-study reproducibility. While the aforementioned reviews provide a broad overview of available utilities, this review addresses a distinct gap by proposing a structured, vertical decision-making roadmap. By emphasizing interoperability, statistical rigor, and FAIR-compliant reproducibility, we provide an executable, end-to-end framework specifically tailored to transform raw lipidomic data into robust biological insights.

Although several comprehensive reviews have summarized lipidomics software ecosystems, most focus on individual tools or specific analytical stages. This review addresses this gap by proposing a structured, decision-driven analytical roadmap that prioritizes interoperability, statistical rigor, and FAIR-compliant reproducibility. A structured, decision-driven integration of preprocessing, statistical modeling, annotation harmonization, and functional inference within a single reproducible R framework remains limited. This review addresses that gap by embedding interoperability, covariate-aware modeling, and enrichment caveats into a unified analytical roadmap. While individual packages such as xcms or lipidr provide extensive documentation for their specific functionalities, a holistic framework that integrates these tools into a validated, end-to-end analytical sequence remains scarce. The present work differentiates itself by providing a decision-making matrix for tool selection and, crucially, offering modular R code) that bridges the gap between disparate packages, ensuring interoperability and statistical rigor across the entire lipidomic lifecycle. This roadmap is specifically designed for bench scientists and clinical researchers with an intermediate understanding of R, aiming to bridge the gap between high-throughput lipidomic data generation and robust statistical interpretation. Ultimately, this roadmap is specifically designed for bench scientists and clinical researchers with an intermediate understanding of R, providing the necessary guidance to transform high-throughput lipidomic data into robust, biologically meaningful insights.

## 2. Computational Infrastructure and Data Formats

The implementation of a robust lipidomics workflow in R necessitates a profound understanding of the computational infrastructure and the underlying data structures that support high-dimensional mass spectrometry (MS) analysis. In this guide, the selection and classification of R packages are based on the previously defined criteria of active maintenance, interoperability, and documentation quality, prioritizing tools that ensure reproducibility and transparency in translational biomedical applications. This selection is intended to serve as a representative baseline for high-performance analysis, though alternative tools may be appropriate depending on specific study designs [6,7].

A primary consideration in lipidomics informatics is the transition from proprietary vendor formats to open-source, standardized data structures. Efficient analysis initiates with the conversion of raw instrument files into accessible formats such as mzML or mzXML, which serve as the universal foundation [17] for downstream processing within the R environment. Conversion from proprietary vendor formats is commonly performed using ProteoWizard’s MSConvert tool prior to R-based processing. Although R has evolved into a scalable analytical framework, its performance is significantly enhanced when leveraging optimized backends such as data.table [18] and Bioconductor S4 infrastructures, like MSnbase [11] and xcms [12].

Beyond domain-specific lipidomics utilities, the infrastructure relies heavily on high-performance data manipulation libraries. The data.table package, for instance, provides the requisite computational speed for handling large-scale files [18], while the tidyverse suite ensures that lipidomic profiles remain structured and compatible across discrete analytical stages [19]. This integration of general-purpose statistical tools with specialized lipidomics packages facilitates a modular pipeline where data flow from raw spectra to refined lipid signatures without the need for manual reformatting. The analytical core of this workflow resides in the capacity to translate processed data into biological significance through rigorous statistical evaluation and visualization. A central component of this transition is the identification of differential lipid abundance, typically represented through high-resolution graphical outputs. The application of volcano plots and differential expression analysis via limma [20] allows for the rapid identification of significant lipid species, effectively bridging the gap between raw computational processing and clinical hypothesis generation. While tools like DESeq2 [21] are fundamentally designed for discrete transcriptomic count data, their variance-stabilizing transformations are occasionally explored in lipidomics for specific normalization purposes; however, for continuous mass spectrometry intensities, linear modeling via limma remains the more statistically appropriate and established choice. All differential testing workflows should incorporate multiple testing correction (e.g., Benjamini–Hochberg FDR control) to mitigate false discoveries inherent to high-dimensional lipidomic data. By maintaining this structured computational approach, researchers ensure that lipidomic findings are not only statistically sound but also fully reproducible across diverse study cohorts and laboratory settings [11,22].

## 3. The Lipidomic Analytical Roadmap

### 3.1. Step 1: Data Acquisition and Pre-Processing

**Figure 1:**
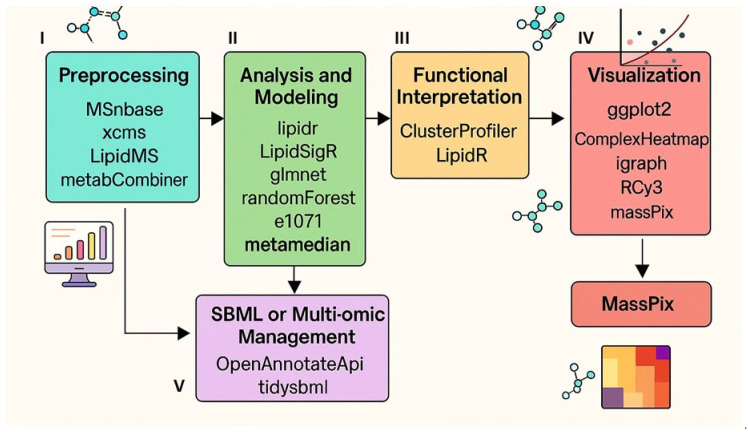
Integrated R-based lipidomics workflow. A schematic overview of the lipidomics pipeline in R, illustrating five key domains (**I**) preprocessing and spectral management, (**II**) multivariate modeling and machine learning, (**III**) functional inference (clusterProfiler, Mendelian Randomization), (**IV**) graph-based network analysis, and (**V**) SBML-compatible multi-omics integration.

To provide clarity and improve readability for this Special Issue, the lipidomics workflow is organized into five discrete analytical stages (Figure 1), reflecting the logical progression from raw data acquisition to functional biological interpretation. Note that complete-case filtering is appropriate only when missingness is assumed MAR; MNAR mechanisms require dedicated imputation strategies (see Section 3.3.3).

#### 3.1.1. Raw Data Handling and Feature Extraction

The initial stage of a lipidomics workflow involves the conversion of complex mass spectrometry (MS) signals into a structured feature table. This process, often referred to as pre-processing, is critical for ensuring that downstream statistical analyses are based on accurate lipid quantification. In the R environment, the xcms package remains the gold standard for processing LC–MS and GC–MS data, providing robust algorithms for peak detection, non-linear retention time correction, and across-sample alignment [10]. Complementary to this, the MSnbase package offers the necessary S4 infrastructure to manage raw MS data and metadata efficiently [11]. While MSnbase excels in data storage and high-level manipulation of spectral metadata, it does not natively perform peak picking or alignment, functions which are the primary strength of xcms [10].

A significant challenge during feature extraction in lipidomics is the co-elution of various ion adducts (such as protonated, sodiated, or ammonium species) and multiply charged ions, which can lead to redundant features and misinterpretation of the lipidome. Although xcms effectively detects individual peaks, it lacks an internal mechanism to group these related signals into a single molecular entity. To address this, researchers must integrate xcms with supplementary packages such as CAMERA [23] or RAMClustR. These tools analyze the correlation between peak shapes and isotopic patterns to identify and group adducts and multiply charged species, effectively collapsing multiple MS signals into a representative feature table. This grouping is essential for complex lipid classes, like cardiolipins or large phospholipids, where multiple ionization states are frequently observed. By leveraging MSnbase [11] for data integrity and xcms [10]—augmented by CAMERA [23]—for feature deconvolution, the workflow ensures a more accurate representation of the biological lipid profile while reducing technical noise [23].

#### 3.1.2. Data Cleaning and Preliminary Wrangling

Before advanced statistical modeling, lipid datasets must undergo rigorous cleaning and quality control. This is primarily achieved through the tidyverse ecosystem [19], which promotes a “tidy” data structure [19] where each variable is a column and each observation is a row. The use of the pipe operator (%>%) from the magrittr [24] package facilitates a readable and reproducible functional style, allowing researchers to chain complex operations such as filtering, log-transformation, and class-level aggregation. High-performance tools like data.table [18] are recommended for handling large-scale cohort studies where file reading speed is a priority.

A key distinction lies in performance: while the tidyverse [19] suite ensures high code readability and reproducibility through its pipe-based syntax, data.table [18] is indispensable for large-scale cohorts (e.g., >1000 samples) where computational speed for file aggregation is a priority.

To bridge the gap between these principles and practical application, a modular R workflow is provided in Supplementary Materials. This implementation (Code S1) demonstrates a standardized pipeline for rapid data ingestion and feature filtration, ensuring data integrity before downstream analysis. Further stabilization is achieved through the transition to specialized LipidomicsExperiment objects, where quality control filters—such as the removal of features with a coefficient of variation (CV) > 30%—and Probabilistic Quotient Normalization (PQN) are systematically applied (Code S2). This structured approach, accessible via the accompanying GitHub repository (version 1.0), allows researchers to execute a complete roadmap from raw feature tables to normalized datasets ready for statistical inference.

#### 3.1.3. Management of Missing Values and Normalization

Missing data are prevalent in untargeted lipidomics due to technical dropouts or biological absence. Advanced imputation techniques are required to prevent bias in downstream multivariate analysis. Packages such as mice (version 3.19.0) [24] and VIM (version 7.0) [25] provide multiple imputation and k-Nearest Neighbors (kNN) algorithms, which are superior to simple mean replacement. Furthermore, the scales package is utilized for data rescaling and normalization, ensuring that lipid intensities are comparable across different analytical batches. These steps are essential for stabilizing the variance and preparing the data for the differential expression analyses shown in later sections. For MNAR-dominated datasets, left-censored imputation approaches (e.g., minimal value replacement or model-based LOD estimation) may be more appropriate than kNN or PMM, which assume local similarity structures.

### 3.2. Step 2: Decision-Making and Package Selection

Before commencing formal statistical inference, the selection of an appropriate computational toolkit is paramount. The lipidomics researcher typically encounters a “fork in the road” based on two criteria: the initial data format (raw spectral files vs. processed feature tables) and the specific biological objective (biomarker discovery vs. mechanistic systems-level interpretation).

The R ecosystem offers a modular architecture, but its efficacy depends on matching the package’s algorithmic strengths to the study’s design. For instance, while xcms [10] is indispensable for peak-picking from raw files, researchers entering the pipeline with peak-picked tables may bypass this step and move directly to lipidr [13] or LipidSigR [26] for downstream analysis.

**Figure 2:**
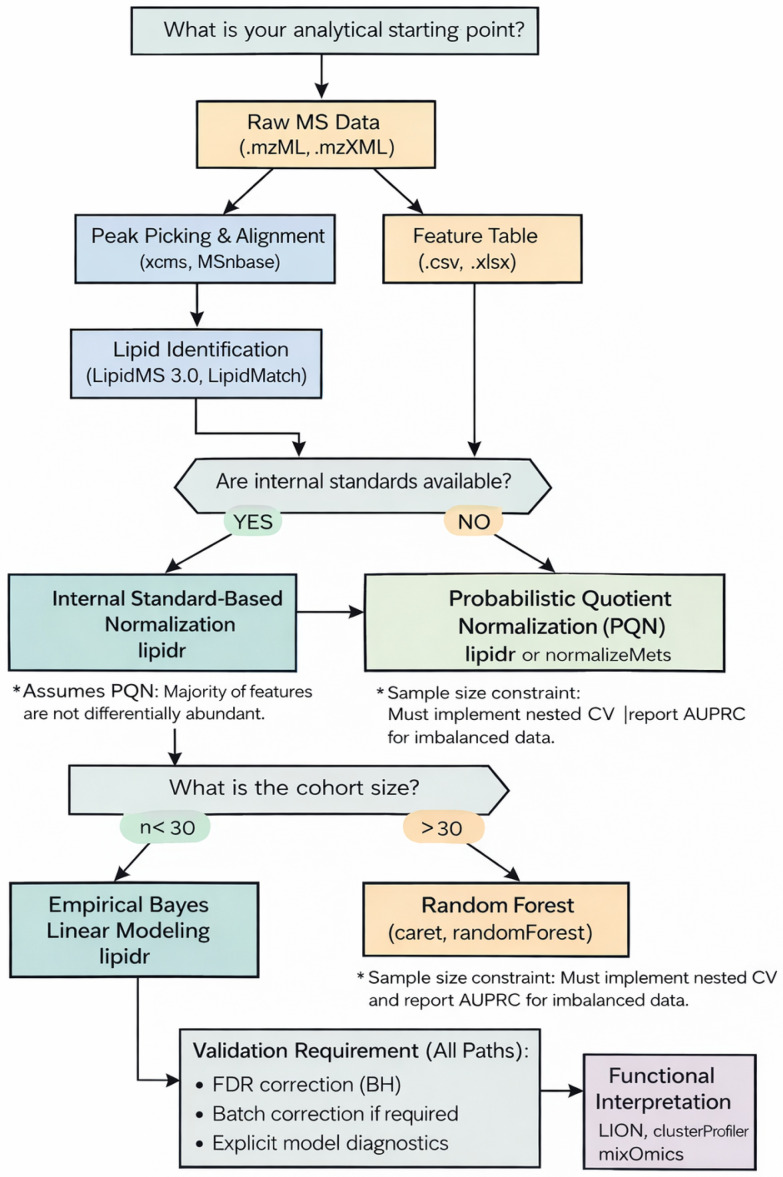
Decision-making matrix for tool selection in lipidomics. The roadmap prioritizes a core set of validated R packages based on their algorithmic strengths: xcms and MSnbase for raw data ingestion, lipidr for standard-based normalization, and a bifurcated statistical path (limma vs. randomForest) dictated by cohort power (*n* < 30 vs. *n* > 30). This logic-driven architecture ensures that tool selection is not arbitrary but statistically grounded.

To streamline this process and ensure methodological consistency, we propose a curated decision-making roadmap (Figure 2: Analytical Logic Flow). This matrix categorizes recommended R packages by their analytical entry points and ultimate study goals, serving as a heuristic guide for constructing coherent and reproducible pipelines. The proposed logic flow operates on three distinct decision nodes: (1) Data Architecture, differentiating between raw MS data handling and processed feature table ingestion; (2) Normalization Strategy, where standard-based normalization via lipidr [13] is prioritized when internal standards are available; and (3) Statistical Modeling, which branches based on cohort size, prioritizing limma [20] for small-sample studies (*n* < 30) and machine-learning frameworks like randomForest [27] for large-scale cohorts (*n* > 30).

The specific R packages highlighted in this roadmap were selected based on three rigorous criteria to ensure a high-performance workflow. First, priority was given to stability and maintenance, focusing on tools with long-term support within the Bioconductor and CRAN repositories to ensure the roadmap remains functional across future R version updates. Second, data interoperability was a primary factor; we selected packages that natively support S4 object structures, such as LipidomicsExperiment and MSnbase, allowing for seamless data transfer between preprocessing, normalization, and functional analysis without the need for manual reformatting. Finally, the inclusion of these specific tools is grounded in methodological benchmarking, reflecting their proven statistical robustness in independent omics studies. While the broader R ecosystem contains specialized libraries for niche applications, this curated selection represents the most reliable, ‘community-standard’ path for reproducible translational lipidomics.

### 3.3. Step 3: Data Cleaning and Quality Control

#### 3.3.1. Quality Control and Signal Drift Correction

Once features have been extracted, the primary challenge is to delineate biological variation from technical stochasticity. A cornerstone of lipidomics best practices is the strategic utilization of Quality Control (QC) samples—typically comprising a pool of all biological samples—injected at regular intervals throughout the LC–MS sequence. Packages such as lipidr [13] and MetaboQC provide automated frameworks for assessing the coefficient of variation (CV) across these QC injections, enabling the systematic filtration of unstable features that exhibit excessive technical variance. Furthermore, for large-scale longitudinal cohorts, the ADViSELipidomics package and the sva infrastructure are indispensable for batch effect correction, ensuring that temporal signal drifts do not confound biological interpretation.

#### 3.3.2. Advanced Normalization Strategies

Normalization is a prerequisite to mitigate variances in sample concentration and injection volume. While total area normalization remains prevalent, lipidomics benefits significantly from internal standard-based normalization. The lipidr package streamlines this by integrating class-specific internal standards [13], effectively correcting for differential ionization efficiencies across diverse lipid categories. For untargeted discovery where standards may be limited, Probabilistic Quotient Normalization (PQN) or LOESS-based normalization via normalizeMets offers a robust alternative to stabilize intra-study variance [28].

#### 3.3.3. Missing Value Imputation and Data Transformation

Lipidomic datasets are inherently characterized by missing values, which are categorized as either Missing At Random (MAR)—due to technical dropouts—or Missing Not At Random (MNAR), typically representing concentrations below the limit of detection (LOD). Simple zero-replacement is statistically discouraged as it artificially deflates variance and biases downstream multivariate analysis.

To address these concerns, our roadmap proposes a two-step decision framework for ensuring data integrity. First, technical quality is prioritized by pruning features with high missing rates (e.g., >20%) or those exhibiting a coefficient of variation (CV) > 30% in pooled QC samples. As demonstrated in Supplementary Code S1, this workflow facilitates the transition to specialized LipidomicsExperiment objects via the lipidr [13] package, ensuring that only stable features are retained.

Second, the remaining missing values are treated based on their suspected mechanism. For MAR data, we advocate for sophisticated imputation frameworks such as k-Nearest Neighbors (kNN) (implemented in the VIM package [25]) or Multiple Imputation by Chained Equations (via the mice package [24]), which leverage local similarity to preserve the original multivariate distribution. Conversely, for MNAR data, left-censored methods (e.g., half-minimum replacement or quantile regression) are more appropriate to account for values below the LOD. This integrated approach ensures statistical consistency, prevents the biases inherent in simple row deletion, and allows for a more representative biological interpretation of the high-dimensional lipidome.

### 3.4. Step 4: Lipid Identification and Structural Annotation

Lipid identification is perhaps the most challenging stage of the computational pipeline, as it requires resolving isobaric species and determining structural features such as chain length, degree of unsaturation, and headgroup specificity. In LC-MS/MS workflows, this process relies on matching experimental fragmentation patterns against simulated or experimental libraries.

#### 3.4.1. Automated Annotation Frameworks and Nomenclature Standards

Lipid identification remains a formidable bottleneck, requiring the resolution of isobaric species and the determination of complex structural attributes. The choice of annotation software often presents a trade-off between algorithmic automation and manual control. The LipidMS 3.0 package provides a state-of-the-art solution by integrating mass distribution and spectral intensities to deliver high-confidence identifications [12], from lipid subclasses down to fatty acid positional isomers. For broader library coverage, LipidMatch [29] utilizes extensive simulated spectra, while LOBSTAHS specializes in identifying lipid species related to oxidative stress through isotopic pattern analysis.

The choice of annotation tool depends on the required structural depth. LipidMS 3.0 [12] excels in rule-based MS/MS identification, offering high confidence in positional isomers. Conversely, LipidMatch [29] provides a more extensive in silico library, making it better suited to broader, untargeted screening, though it requires more rigorous manual validation to avoid false positives. The effectiveness of these tools is heavily dictated by the acquisition mode and fragmentation depth. While most R-based frameworks, including LipidMS, are optimized for Data-Dependent Acquisition (DDA) at the MS2 level, the processing of Data-Independent Acquisition (DIA) data (e.g., SWATH) often requires external deconvolution, such as MS-DIAL, before R-based downstream analysis [30], as native R support for complex DIA-multiplexed spectra remains a developing area. Furthermore, while MS3 data can provide definitive headgroup and backbone confirmation, current R packages primarily utilize MS2 fragmentation as the standard operational baseline for high-throughput annotation.

The choice of annotation tool also depends on the fragmentation technology and the required structural depth. LipidMS 3.0 excels in rule-based identification, which is particularly advantageous when dealing with different techniques such as Collision-Induced Dissociation (CID), Higher-energy Collisional Dissociation (HCD), or advanced Electron Activation Dissociation (EAD). Because rule-based engines can be tailored to specific fragmentation patterns, they offer superior depth in resolving double-bond positions and sn-positions compared to static libraries [31]. In contrast, LipidMatch [29] provides a more extensive in silico library and uniquely supports the integration of custom spectral libraries, which is essential for researchers targeting novel or niche lipid species. For datasets enriched in structurally complex lipids, such as oxidized species or oxylipins, LOBSTAHS specializes in identifying these biomarkers through rigorous isotopic pattern and adduct hierarchy analysis. This specialized approach is necessary for clinical projects where simple molecular-level identification (e.g., PC 32:2) is insufficient, and precise information regarding oxygen insertion or fatty acyl chain modification is required.

Crucially, to ensure cross-study comparability and data longevity, all identifications must adhere to internationally recognized Nomenclature Standards. Specifically, rgoslin [32] provides a high-performance parser that translates disparate and often inconsistent lipid naming strings into a singular, grammar-based format that aligns strictly with the LIPID MAPS international classification system [4]. The integration of the LIPID MAPS classification system is paramount, as it provides a hierarchical structure and standardized identifiers (LM_IDs) that resolve ambiguities in lipid naming. Adherence to these standards, supported by tools like rgoslin [32], facilitates the seamless translation of experimental outputs into biologically interoperable data, significantly enhancing the reliability and meta-analysis potential of findings in large-scale clinical studies.

#### 3.4.2. Structural Feature Extraction

Once lipids are named, packages like lipidr [13] and LipidSigR [26] are essential for decomposing these names into structural metadata (e.g., total carbon count and double bond content). This allows researchers to perform analyses not just on individual molecules, but on structural patterns. This systematic annotation is a prerequisite for the functional enrichment and network analyses discussed in subsequent sections.

Lipidr [13] is optimized for seamless integration with S4 experiment objects and automated enrichment, whereas LipidSigR offers a more user-friendly, ‘all-in-one’ graphical interface for rapid dimensionality reduction and visualization. For researchers embedded in the Bioconductor workflow, lipidr [13] offers greater interoperability.

### 3.5. Step 5: Diversity and Differential Analysis

After successful identification and rigorous quality control, the workflow shifts toward characterizing the heterogeneity of the lipidome and identifying significant biomarkers.

#### 3.5.1. Lipidome Diversity and Heterogeneity

Inspired by microbial ecology, diversity analysis provides quantitative insights into the “richness” (number of species) and “evenness” (relative abundance) of lipid compositions. The vegan package enables alpha-diversity metrics (e.g., Shannon index) and beta-diversity ordination using NMDS with Bray–Curtis dissimilarity [33]. For spatial lipidomics, tools like massPix [34] and Cardinal [35] extend these concepts to Mass Spectrometry Imaging (MSI), enabling the assessment of intra-tissue heterogeneity and spatial clustering. Importantly, the imputation strategy must align with the assumed missingness mechanism (MAR vs. MNAR). Applying kNN to MNAR-dominated datasets may introduce artificial similarity structures.

As detailed in  Supplementary Materials (Code S3), this ecological framework allows for the visualization of sample clustering and the identification of group-specific lipidomic signatures based on compositional similarity. This implementation demonstrates how raw lipid matrices are transformed into ordination plots, providing a high-level overview of the lipidome’s complexity.

#### 3.5.2. Differential Abundance and Biomarker Discovery

The core of most lipidomics studies is the identification of lipids that vary significantly between experimental conditions. The lipidr package streamlines this through an integrated framework for *t*-tests, ANOVA [13], and multivariate modeling like PLS-DA. For complex clinical datasets where batch effects or multi-omics integration are required, limma and mixOmics provide robust linear modeling and supervised classification methods [8]. As identified in our Analytical Logic Flow (Figure 2), the choice between these tools is governed by statistical power; while limma utilizes empirical Bayes moderated linear models to stabilize variance in small-sample cohorts (*n* < 30), multivariate models like PLS-DA via mixOmics are better suited to uncovering structured variation in larger datasets. As part of this analytical roadmap, we demonstrate the execution of differential abundance testing and the subsequent prioritization of candidate biomarkers in Supplementary Materials (Code S4). This implementation utilizes lipidr to calculate statistical significance and generate high-resolution volcano plots, which map the magnitude of change (log_2 Fold Change) against statistical significance (−log_{10} *p*-value), facilitating the rapid identification of biologically relevant lipids.

Beyond traditional statistics, advanced biomarker discovery is increasingly supported by machine learning. The roadmap incorporates penalized regression via glmnet (4.1.8) [36] (LASSO/Elastic Net) for feature selection and ensemble learning through randomForest (4.7.1.2) [27] to capture non-linear relationships. The BioPred package (via XGBoost v1.7.5) [37] employs Extreme Gradient Boosting (XGBoost) to identify predictive lipid signatures, while the e1071 package (1.7.16) further extends these capabilities by providing Support Vector Machine (SVM) classifiers for robust group separation [38]. When deploying machine learning via BioPred [37] or mixOmics (6.24.0) [9], rigorous validation is mandatory to prevent over-optimism. Given the typically high feature-to-sample ratio in lipidomics, we emphasize the use of nested cross-validation to decouple model selection from performance estimation. Furthermore, in clinical cohorts with class imbalance (e.g., rare diseases), metrics such as the Area Under the Precision-Recall Curve (AUPRC) should be prioritized over simple accuracy to avoid data leakage and ensure model generalizability. As a practical guideline, the feature-to-sample ratio should ideally remain below 10:1 in supervised models unless dimensionality reduction or penalized regression is applied.

#### 3.5.3. Statistical Assumptions and Model Selection

The validity of differential abundance testing in lipidomics depends directly on the careful verification of the underlying biostatistical assumptions. Because MS-based datasets are inherently high-dimensional, the analytical approach cannot be applied mechanically. Instead, a structured model selection process must be followed in order to ensure that the detected differences truly reflect biological variation and not statistical artifacts.

Before applying parametric tests, the effect of data transformation (e.g., log_2_ or Generalized Log) should first be evaluated. The assessment of normality should not be performed on the raw distributions of each individual feature, since large-scale testing can artificially increase rejection rates. Instead, normality must be examined at the level of model residuals, which provides a more reliable indication of whether parametric assumptions are satisfied [15]. If certain lipid features continue to deviate substantially from normality despite transformation, non-parametric alternatives such as the Wilcoxon rank-sum test or kruskal.test should be applied, thereby reducing the risk of inflated Type I errors. Parametric models such as ANOVA additionally assume equal variance across experimental groups. For this reason, homoscedasticity should be formally assessed using tests such as Levene’s or Bartlett’s. When heteroscedasticity is detected, appropriate adjustments are required. Weighted linear models or variance-stabilizing transformations (VST), as implemented in packages such as DESeq2 [21] or limma (via voom) [20], can be used to preserve statistical power while maintaining model validity.

Regarding differential testing, limma (via the voom transformation) is generally preferred over DESeq2 for lipidomic intensities. While DESeq2 is the authority for count-based RNA-seq data, limma’s empirical Bayes shrinkage is better suited to the continuous, log-normal distribution of mass spectrometry data, providing more stable variance estimation in small clinical cohorts [20,21].

In many experimental settings, lipid abundance is influenced not only by the primary condition of interest but also by confounding variables such as age, sex, BMI, or batch effects. To isolate the biological signal of interest, multifactorial linear modeling should therefore be applied. The limma framework is particularly suitable in this context, as it allows both continuous and categorical covariates to be incorporated into the design matrix through model.matrix [20]. In this way, the resulting lipid signatures can be interpreted as independently associated with the experimental factor under investigation. The practical implementation of this covariate-adjusted modeling strategy, including construction of the design matrix, is provided in the Supplementary Materials (Code S4). Finally, given that hundreds or even thousands of lipid species are tested simultaneously, the probability of false discoveries increases substantially. Adjustment for multiple testing is therefore not optional but essential. The Benjamini–Hochberg (BH) False Discovery Rate (FDR) correction remains the standard approach in lipidomics for controlling Type I error rates [12,28]. A stringent threshold (e.g., q < 0.05) is recommended to ensure that candidate biomarkers selected for downstream functional validation are statistically robust and biologically credible.

### 3.6. Step 6: Functional Interpretation and Enrichment Analysis

A critical challenge in lipidomics is that lipids are not direct gene products. To perform enrichment analysis via clusterProfiler [15], we implement a ‘protein-centric’ mapping approach. As illustrated in Supplementary Materials (Code S5), significant lipids are first mapped to their regulatory enzymes (e.g., desaturases, phospholipases) using the LION ontology [39] or BridgeDb. This enzyme list then serves as the input for GO/KEGG enrichment. We caution researchers that this mapping assumes that changes in lipid abundance directly reflect enzyme activity, a proxy that must be interpreted with biological nuance. Importantly, enrichment results derived from lipid-to-enzyme mapping should be interpreted as hypothesis-generating rather than confirmatory, since lipid abundance does not necessarily equate to enzyme activity.

#### 3.6.1. Lipid Ontology and Pathway Mapping

Unlike transcriptomics, where gene symbols are standardized, lipidomics requires specialized ontologies that account for structural hierarchies. The lipidr package facilitates enrichment analysis by utilizing the Lipid Ontology (LION) framework, allowing researchers to assess whether specific lipid classes or structural features such as chain length or unsaturation level are overrepresented [39] in their results. For broader pathway analysis, clusterProfiler remains the premier tool for conducting Gene Ontology (GO) and KEGG pathway enrichment [15], particularly when lipidomics data are integrated with proteomic or transcriptomic profiles. Functional interpretation offers two distinct paths: LION performs lipid-centric enrichment directly on structural features (e.g., saturation, chain length), avoiding mapping biases. In contrast, clusterProfiler enables gene-centric pathway analysis (KEGG/GO) by mapping lipids to regulatory enzymes, making it the tool of choice for multi-omic integration studies [15,39].

#### 3.6.2. Multi-Omics Integration and Network Analysis

To achieve a systems-level understanding, lipids must be linked to their regulatory enzymes and transporters. The biomaRt package is used to map lipid-related proteins to genomic identifiers [40], enabling the construction of cross-omics networks. For complex interactions, mixOmics provides advanced multivariate methods like DIABLO, which identifies correlated signatures across different omics layers [9], providing a holistic view of the disease state.

This mechanistic transition is practically demonstrated in Supplementary Materials (Code S5), where we implement functional enrichment analysis using the clusterProfiler package. By mapping significant lipids to their associated biological processes and gene sets, this workflow identifies enriched metabolic pathways, offering a high-resolution view of the cellular state through automated dotplot visualizations. Furthermore, the tidysbml package [41] allows for the extraction of biological interaction data into R, which can then be visualized as functional networks using igraph or RCy3, bridging the gap between molecular abundance and systems biology.

## 4. Best Practices for Reproducibility in Downstream Computational Lipidomics

Achieving computational reproducibility in downstream lipidomics analysis requires rigorous control over the computational environment. Given the dynamic nature of R, where package updates can alter algorithmic behaviors, the use of environment management tools like renv is essential. This creates isolated project libraries that “lock” specific versions of dependencies, ensuring that an analysis performed with specific versions of xcms [10] or lipidr [13] yields identical results in the future [22] (Table S1).

**Figure 3:**
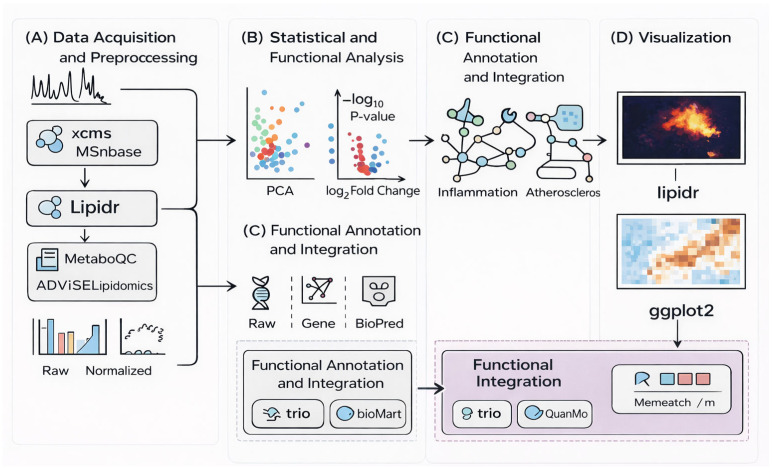
Functional prediction, routine data analysis, and visualization in R. The workflow begins in panel (**A**) with raw data preprocessing using xcms, MSnbase, and lipidr, where normalization and quality control establish a reliable analytical baseline. In panel (**B**), statistical analysis (e.g., PCA, differential testing) is performed to identify structured variation and significant lipid features. Panel (**C**) maps these findings (derived from either statistical analysis or data preprocessing) to biological context through functional annotation and pathway integration. Finally, panel (**D**) summarizes and communicates the results through visualization. The arrows indicate the logical progression from data acquisition to biological interpretation, with the possibility of iterative refinement between stages.

In parallel, the adoption of literate programming frameworks, such as R Markdown or Quarto, facilitates the integration of raw code with biological documentation. Through these tools, the generation of complex outputs, such as the volcano plots in Figure 3, becomes a traceable roadmap from raw data to final representation. Ultimately, adhering to “Tidy Data” principles and providing detailed in-line documentation for parameter choices—such as peak-picking thresholds—constitutes the core of ethical and scientifically valid research [42].

Beyond environment locking, the standardization of metadata is a critical pillar of reproducibility. Integrating standardized nomenclature, such as the grammar provided by GOSLIN [32], ensures that lipid identifications are consistent across datasets. This level of detail allows for the seamless transition of workflows between different laboratory settings, effectively mitigating the “reproducibility crisis” in high-throughput omics. By embedding these parameters directly into the S4 objects provided by engines like MSnbase [11], the data becomes self-documenting and easier to audit during the peer-review process [7].

Finally, the democratization of lipidomics data necessitates the use of open-source repositories and version control systems. By hosting R scripts and documentation, the lipidomics community can foster a culture of collaborative peer-review for computational pipelines [7,15]. This shift toward Open Science ensures that the modular tools—from functional enrichment with clusterProfiler [15] to multi-omic integration via tidysbml [41]—remain accessible, verifiable, and adaptable for future clinical discovery. Table 1 summarizes the most important R packages in lipidomic research, providing a curated overview of the tools that facilitate this reproducible and high-performance analytical journey. Containerization (e.g., Docker) can further enhance reproducibility by preserving system-level dependencies beyond R package versions.

### Roadmap Validation: A Case Study Application

The intrinsic value of any computational framework is ultimately judged by its empirical validation in practice. Therefore, rather than limiting this work to a theoretical presentation of package capabilities, this section illustrates the application of the proposed framework through a representative case study. To ensure maximum transparency and technical rigor, the entire analytical sequence is implemented in Supplementary Materials (Code S6). This code serves as a functional template, allowing researchers to follow, step-by-step, the transition from raw intensity matrices to high-confidence biomarkers [7,15].

In this illustrative application, the integrated workflow was applied to a high-fidelity synthetic dataset designed to simulate the lipidomic profile of human plasma, strictly adhering to international IUPAC nomenclature [32]. Following the Analytical Logic Flow (Figure 2), the first critical milestone achieved was the technical stabilization of the data via Probabilistic Quotient Normalization (PQN), as described in Step 2 of the roadmap [28]. The implementation of PQN was selected over standard-based methods due to the untargeted nature of the study, offering a robust approach to balance variations arising from technical errors or sample dilution effects. In practice, this normalization led to a noticeable reduction in the intra-group Coefficient of Variation (CV) compared to the initial raw values, effectively stabilizing the lipidome profile. This noise reduction is essential, as it enables the detection of subtle biological signals that would otherwise remain obscured by the stochastic variability of analytical instruments [13,17].

In alignment with our decision matrix for small-sample cohorts (*n* < 30), the utility of the roadmap was further demonstrated through the statistical prioritization of lipids using the direct integration of limma (empirical Bayes moderated linear model), as analyzed in Step 4 of Code S6 [20,33]. By leveraging established linear modeling instead of “black-box” solutions, the workflow facilitates reliable variance estimation, even in cases with limited sample sizes. The results are encapsulated in the Volcano Plot, which clearly delineates lipids with statistically significant alterations (*p* < 0.05) and substantial biological effect size (|log_2 FC| > 1), thereby managing the probability of false-positive discoveries [21,37].

Ultimately, this application demonstrates that the transition to S4 container structures (via the as_lipidomics_experiment function) serves as a key step for data integrity [6,11]. This architecture allows for immediate functional enrichment analysis through specialized ontologies such as LION, without requiring manual data reformatting [39]. In conclusion, the implementation in Code S6 illustrates that a structured, decision-driven integration of R packages can transform fragmented data into a cohesive and methodologically sound sequence for lipidomics research [12,26].

## 5. Common Pitfalls in R-Based Lipidomics

Despite the robustness of the R ecosystem, lipidomic datasets remain inherently complex, and this complexity can easily lead to systematic errors in interpretation. For this reason, particular attention must be given to avoiding methodological pitfalls that may compromise the reliability of clinical or biological conclusions. One frequent mistake concerns the handling of missing values. In mass spectrometry data, missing measurements often reflect concentrations below the limit of detection (MNAR) rather than a true absence of a lipid species. Replacing these values with zeros artificially reduces variance and may generate misleading statistical significance. Instead, missing values should be handled using structured imputation approaches, such as k-nearest neighbors (kNN) or Multiple Imputation, in order to preserve the multivariate structure of the lipidome [24,25].

Another critical issue arises with supervised classification methods such as Partial Least Squares Discriminant Analysis (PLS-DA). Although widely applied for group discrimination, PLS-DA is particularly sensitive to overfitting. Reporting separation plots without proper cross-validation (e.g., Q^2^ statistics) or permutation testing substantially weakens the validity of the conclusions. Supervised approaches should therefore be implemented only within a rigorous validation framework, as supported by tools such as mixOmics [9]. Batch effects represent an additional and often underestimated source of distortion in large-scale lipidomics studies. When samples are processed across multiple analytical runs, signal drift and inter-run variability may obscure true biological signals or introduce artificial clustering. For this reason, correction methods such as ComBat (implemented in the sva package) or ADViSELipidomics should be applied prior to downstream statistical analysis to ensure comparability across batches. Consistency in lipid nomenclature is equally important. The use of non-standardized names (for example, “PC 34:1” versus “PC(16:0/18:1)”) complicates database mapping and limits cross-study reproducibility. Harmonizing lipid names with tools such as rgoslin ensures accurate structural parsing and prevents errors during feature annotation and biological interpretation [32].

From a statistical standpoint, reporting unadjusted *p*-values in high-dimensional lipidomics experiments is methodologically inappropriate. Because hundreds of lipid species are typically tested simultaneously, the probability of Type I errors is substantial. Therefore, False Discovery Rate (FDR) correction, such as the Benjamini–Hochberg procedure, must be systematically applied in all differential abundance analyses [12,20]. Finally, in functional enrichment analysis, careful definition of the background universe is essential. The appropriate reference set should consist only of the lipids that were actually detected and quantified in the experiment, rather than the entire known lipidome. Failure to define this correctly can inflate pathway significance and lead to biased biological interpretation, particularly when using tools such as clusterProfiler [15,28].

## 6. Conclusions

The findings of this work underscore that R has evolved into a robust and mature computational ecosystem, providing a comprehensive suite of interoperable tools for the lipidomics community. The integration of preprocessing standards such as xcms [10,43] and LipidMS 3.0 [12] with specialized quality control frameworks like lipidr [12] and ADViSELipidomics has established a reliable foundation for LC–MS workflows [12,32]. However, as this review highlights, the primary challenge remains the fragmentation of the computational landscape. While individual packages excel at specific analytical stages, the responsibility often falls on the researcher to assemble multi-package pipelines, which can introduce variability and complicate the standardization of end-to-end workflows. A critical challenge identified in current lipidomics research is the interoperability of lipid annotations. The variability in lipid naming conventions across different platforms and software tools poses a significant risk to the consistency of downstream biological interpretations. The emergence of community-driven standards and the development of tools like rgoslin [32] are pivotal in addressing this issue, as they facilitate the translation of diverse nomenclature into LipidMaps-compatible formats [4]. Ensuring that lipid identifiers are harmonized is not merely a technical requirement but a prerequisite for reproducible cross-platform comparisons and meta-analyses [15,17].

Looking forward, the evolution of lipidomics data analysis will be shaped by the continued integration of machine learning and causal inference tools. As demonstrated by the inclusion of packages like BioPred [37] and TwoSampleMR, R provides a highly integrated and mature ecosystem for lipidomics, particularly within the Bioconductor infrastructure to bridge the gap between descriptive lipidomics and predictive clinical modeling [26,34]. Furthermore, the capacity of R to handle multi-omics integration through frameworks like mixOmics and DIABLO [9] provides a distinct advantage over general-purpose programming languages or GUI-only solutions. These capabilities support the development of flexible, scalable, and—most importantly—reproducible analyses that can be shared and validated across the global scientific community. In conclusion, R provides a comprehensive and transparent environment that supports the entire lipidomics analytical lifecycle. By leveraging an integrated roadmap of packages—spanning from raw data processing with MSnbase to functional enrichment with clusterProfiler—researchers can translate complex lipidomic profiles into meaningful biomedical insights. Continued progress in harmonizing data structures coupled with the widespread adoption of reproducible pipelines will reinforce R’s role as a central platform for lipidomics, ultimately accelerating the translation of lipidomic signatures into clinical applications and therapeutic targets. Nevertheless, this roadmap does not replace dataset-specific benchmarking as optimal package selection may vary with instrumentation, acquisition mode and study design.

## Figures and Tables

**Table 1:** Overview of major R tools used in lipidomics, outlining their core functions, limitations and challenges.

Category	R Package	Core Functions	Limitations & Constraints	Refs.
Preprocessing	xcms (3.22.0)	Peak detection, alignment, filtering	High computational cost; steep learning curve for parameter optimization.	[10]
	MSnbase (2.26.0)	Spectra management, S4 infrastructure	Primarily designed for proteomics; requires custom scripts for complex lipidomics.	[11]
	lipidMS (3.0.0)	MS/MS identification & annotation	Identification is heavily dependent on the quality of fragmentation libraries.	[12]
Analysis and Modeling	lipidr (2.14.1)	Univariate/Multivariate analysis, Volcano plots	Limited flexibility for complex multi-factorial longitudinal study designs.	[13]
	LipidSigR (1.0.0)	All-in-one analysis, PCA, clustering	Newer package; smaller community support compared to established tools.	[26]
	mixOmics (6.24.0)	Multi-omics integration (DIABLO)	Risk of overfitting in small sample cohorts; requires rigorous cross-validation.	[9]
	limma (3.56.2)	Moderated linear models (small cohorts)	Assumes log-normal distribution; requires voom transformation for count-like data.	[20]
	glmnet (4.1.8)	Penalized regression (LASSO/Elastic Net)	Linear assumptions; may struggle with highly non-linear lipidomic patterns.	[36]
	randomForest (4.7.1.2)	Ensemble learning, feature importance	“Black-box” nature makes biological interpretation of individual features difficult.	[27]
Functional Interpretation	clusterProfiler (4.8.1)	GO/KEGG enrichment analysis	Lipid-to-Gene mapping can introduce bias if the background universe is poorly defined.	[15]
	LION (v1.0)	Lipid-specific ontology enrichment	Limited by the current depth of lipid-specific functional annotations.	[39]
Visualization	ComplexHeatmap (2.16.0)	Multi-dimensional heatmaps	High memory consumption for very large datasets (>10,000 features).	[14]
	ggplot2 (4.0.2)	Publication-grade plots	Requires extensive coding for non-standard, complex multi-panel figures.	[42]
	e1071 (1.7.16)	SVM classification and visualization	Sensitive to parameter tuning (sigma/cost); prone to overfitting without CV.	[38]

## Data Availability

No new data were created or analyzed in this study.

## Supplementary Materials

The following supporting information can be downloaded at: https://www.mdpi.com/article/10.3390/metabo16050288/s1, Table S1: R Package Selection Strategy and Technical Documentation. Code S1: Preprocessing; Code S2: QC & Normalization; Code S3: Diversity; Code S4: Statistics; Code S5: Functional Analysis; Code S6: Reproducible R Workflow for Roadmap Validation. The R scripts and computational workflows developed for this roadmap are openly available in the https://github.com/MariaChristinaPapatheodorou/Computational-Roadmap-for-Lipidomics-in-R-From-Raw-Data-to-Functional-Insight---codes repository (accessed on 15 April 2026).
