# Supplementary material for: A Structured Computational Roadmap for Lipidomics in R: Reproducible Workflows from Raw Data to Functional Insight
Source: Metabolites. 2026 Apr 22;16(5):288. doi: 10.3390/metabo16050288 (PMC13208769; doi:10.3390/metabo16050288)
Supplement: Supplementary file 1 [file metabolites-16-00288-s001.zip › metabolites-4226598-supplementary.pdf]

Supplementary Material

A Structured Computational Roadmap for Lipidomics in R: Reproducible Workflows from Raw Data to Functional Insight

Maria-Christina P. Papatheodorou, Panagiotis Vlamos and Marios G. Krokidis*

* Correspondence: mkrokidis@ionio.gr

GitHub Repository: GitHub Link

Software and Versions:

R Programming Language: Version 4.3.0 (2023-04-21 ucrt).

Integrated Development Environment (IDE): RStudio 2023.09.0 Build 463 "Desert Sunflower" for Windows.

Platform: x86_64-w64-mingw32/x64 (64-bit).

Key Packages: lipidr, clusterProfiler, xcms, MSnbase, tidyverse, vegan.

Code Modules Overview:

Code S1 (Preprocessing): Implementation of a reproducible data cleaning and exploratory visualization pipeline. This script outlines a standardized workflow for the initial processing of lipidomic datasets using the tidyverse ecosystem. The pipeline initiates with high-speed data ingestion via the readr package, followed by a sequential series of operations linked by the pipe operator (%>%). Key steps include: 1.Feature Filtration: The removal of incomplete observations (NAs) to maintain data integrity. 2.Variance Stabilization: Application of a log_{10} transformation to mitigate heteroscedasticity and normalize the intensity distribution. 3. Class-level Aggregation: Utilizing group_by and summarise to collapse individual lipid species into broader biological classes for trend identification.

```
# CAUTION: Simple filtering of NAs assumes MAR; for MNAR data, consider quantile regres sion or LOD-based imputation.

# Load core suites for lipid data wrangling

library(readr)  # Fast data import

library(dplyr)  # Data manipulation
```

```
library(ggplot2) # Visualization
# Step 1: Import lipidomics dataset
lipid_data <- read_csv("lipidomics_dataset.csv")
# Step 2: Quality control and Advanced Imputation (Roadmap Decision Framework)
# 1. Filter out features with more than 20% missing values (Pruning)
missing_threshold <- 0.2
lipid_data_filtered <- lipid_data %>%
  filter(rowSums(is.na(.)) / ncol(.) <= missing_threshold)
# 2. Advanced Imputation instead of simple deletion (na.omit is avoided)
# We use kNN for MAR data as recommended in the manuscript
if (!requireNamespace("impute", quietly = TRUE)) install.packages("impute")
library(impute)
# Convert to matrix for imputation
mat_data <- as.matrix(lipid_data_filtered[,-1]) # Assuming first col is Lipid Name
imputed_mat <- impute.knn(mat_data)$data
# 3. Variance Stabilization and Class-level Aggregation
clean_data <- as.data.frame(imputed_mat) %>%
  mutate(log_intensity = log10(. + 1)) # Mitigate heteroscedasticity
# Step 3: Visualization of class-level distribution
ggplot(clean_data, aes(x = lipid_class, y = mean_log_intensity, fill = lipid_class)) +
geom_bar(stat = "identity") +
theme_minimal() +
labs(title = "Average Lipid Intensity per Class",
x = "Lipid Class", y = "Mean log10(Intensity)") +
theme(axis.text.x = element_text(angle = 45, hjust = 1))
```

Code S2 (QC & Normalization): Quality control filtering and normalization pipeline using specialized lipidomics packages. This implementation demonstrates the transition from raw feature tables to a normalized LipidomicsExperiment object. The script utilizes lipidr

to execute a rigorous QC filter, removing features with a CV > 30% in pooled QC samples to eliminate unstable variables. Subsequently, it applies Probabilistic Quotient Normalization (PQN) to stabilize the dataset against concentration-induced variations. The workflow concludes with a boxplot-based assessment (plot_samples), providing a visual confirmation of variance stabilization across the sample cohort.

```
# Example of QC filtering and normalization using lipidr

# Input: Tidy data frame with 'Lipid', 'Sample', and 'Intensity' columns. lipidr converts this

into a LipidomicsExperiment object, inheriting from SummarizedExperiment.

library(lipidr)

# Create a LipidomicsExperiment object (assuming data is already tidied)

lipid_ex <- as_lipidomics_experiment(clean_data)

# Filter lipids with high CV in QC samples (> 30%)

#

lipid_ex inherits from SummarizedExperiment, enabling compatibility with

Bioconductor workflows

lipid_ex <- filter_low_cv(lipid_ex, measure = "Area", threshold = 0.3)

# Perform PQN normalization

lipid_ex_norm <- normalize_pqn(lipid_ex, measure = "Area")

# Visualizing the effect of normalization

plot_samples(lipid_ex_norm, measure = "Area", type = "boxplot")
```

Code S3 (Diversity): Calculation of diversity metrics and ordination. This implementation leverages the vegan package to quantify lipidome diversity. The Shannon index provides a measure of molecular richness, while the NMDS ordination visualizes sample clustering based on compositional similarity, offering a high-level view of group-specific lipidomic signatures.

```
library(vegan)

# Calculate Shannon diversity index to assess lipidome richness

diversity_index <- diversity(lipid_matrix, index = "shannon")

# Perform NMDS ordination to visualize sample clustering
```

```
ordination <- metaMDS(lipid_matrix, distance = "bray")
plot(ordination, main = "NMDS of Lipidomic Profiles")
```

Code S4 (Statistics): Differential Abundance with Covariate Adjustment. This module demonstrates the transition from simple comparisons to robust linear modeling. By utilizing a design matrix, we account for potential confounders and apply FDR correction to identify high-confidence biomarkers.

```
# Note: We prioritize limma over count-based tools (like DESeq2)
# because lipidomic intensities are continuous variables, not discrete counts.
library(lipidr)
library(limma)
# lipidr leverages the empirical Bayes framework of limma to stabilize variance and
increase statistical power.
# 1. Define Design Matrix (Accounting for Group and Batch/Covariates)
# Assuming 'Group' is our main condition and 'Batch' is a confounder
design <- model.matrix(~ Group + Batch, data = colData(lipid_ex_norm))
# 2. Perform Differential Analysis using a Linear Model framework
# The 'de_analysis' function in lipidr wraps limma for robust inference
de_results <- de_analysis(
lipid_ex_norm,
design = design,
coef = "GroupTreatment" # Specify the comparison of interest
)
# 3. Apply Multiple Testing Correction (FDR/Benjamini-Hochberg)
# lipidr performs this internally, but we can extract and filter
significant_lipids <- de_results %>%
filter(adj.P.Val < 0.05 & abs(logFC) > 1)
# 4. Visualization: Enhanced Volcano Plot
# Mapping Log2 Fold Change vs -Log10 Adjusted P-value
```

```
plot_results(de_results, type = "volcano", p_threshold = 0.05)

# Empirical Bayes shrinkage improves variance estimation in small sample sizes
```

Code S5 (Functional Analysis): Implementation of functional enrichment analysis. This implementation demonstrates the use of clusterProfiler to identify enriched biological processes. By mapping significant lipids to their associated gene sets, the workflow provides a mechanistic view of the cellular state through a high-resolution dotplot.

```
library(clusterProfiler)

# For lipid-specific functional analysis, we recommend mapping lipids to

# LION terms or KEGG pathways using the lipidr::enrich_lipid_classes() function.

# Functional enrichment analysis using lipid-related gene sets

enrich_result <- enrichGO(gene = significant_genes,

OrgDb = org.Hs.eg.db,

ont = "BP",

pAdjustMethod = "BH")

# Visualize the top enriched biological processes

dotplot(enrich_result, showCategory = 15) +

ggtitle("Functional Enrichment of Lipid Metabolism Pathways")
```

Code S6 (Reproducible R Workflow for Roadmap Validation.): This executable script demonstrates the end-to-end application of the proposed roadmap, using a synthetic dataset to ensure analytical transparency and computational reproducibility. The workflow integrates data ingestion into S4 structures (Step 1), variance stabilization via Probabilistic Quotient Normalization (Step 2), and rigorous biomarker discovery using an empirical Bayes moderated linear model (Step 4). By providing a direct link between raw data processing and high-fidelity visualization (Step 6), this case study validates the interoperability of the integrated R-packages and serves as a benchmark for standardized lipidomic data analysis.

```
# ------------------------------------------------------------------------

# VALIDATION CASE STUDY: Integrated Lipidomics Roadmap

# Description: This script demonstrates the practical application of the
```

```
# roadmap using a synthetic dataset to ensure full reproducibility.
# Introducing synthetic missing values to demonstrate roadmap robustness
intensities[sample(1:80, 5)] <-NA
# Proceed with the roadmap's imputation step as described in Section 3.3.3
# ----------------------------------------------------------------------
# 1. Load Required Libraries
library(lipidr)
library(ggplot2)
library(limma) # Direct limma integration for maximum stability
# 2. Data Ingestion & Formatting (Roadmap Step 1)
# Generating a synthetic dataset with LION-compliant lipid nomenclature
set.seed(123)
lipids <- c("PC(32:0)", "PC(34:1)", "PC(36:2)", "PE(32:0)", "PE(34:1)",
"TG(50:1)", "TG(52:2)", "TG(54:3)")
intensities <- matrix(runif(80, 100, 1000), nrow = 8)
colnames(intensities) <- paste0("Sample_", 1:10)
df_input <- data.frame(Molecule = lipids, intensities)
# Convert to LipidomicsExperiment S4 object (Standard Bioconductor architecture)
li_exp <- as_lipidomics_experiment(df_input)
# 3. Quality Control & Normalization (Roadmap Step 2)
# Applying Probabilistic Quotient Normalization (PQN) to reduce technical variance
li_exp_norm <- normalize_pqn(li_exp, measure = "Area")
# 4. Statistical Prioritization & Biomarker Discovery (Roadmap Step 4)
# Direct implementation of the Linear Models for Microarrays (limma) engine
# extracting the normalized matrix for high-performance testing
mat <- assay(li_exp_norm)
group <- factor(rep(c("Control", "Disease"), each = 5))
# Constructing the Design Matrix and applying empirical Bayes moderation
```

```
design <- model.matrix(~ group)
fit <- lmFit(log2(mat + 1), design) # Log2 transformation for variance stabilization
fit <- eBayes(fit)
# Extracting statistical metrics for the prioritizing significant species
df_results <- topTable(fit, coef = 2, number = Inf)
df_results$Molecule <- rownames(df_results)
# 5. Data Visualization (Roadmap Step 6)
# Generating a volcano plot to differentiate biological from statistical significance
ggplot(df_results, aes(x = logFC, y = -log10(P.Value))) +
geom_point(aes(color = P.Value < 0.05), size = 3) +
geom_vline(xintercept = c(-1, 1), linetype = "dashed", color = "darkgrey") +
geom_hline(yintercept = -log10(0.05), linetype = "dashed", color = "darkgrey") +
scale_color_manual(values = c("black", "red")) +
theme_minimal() +
labs(title = "Roadmap Validation: Differential Abundance",
subtitle = "Calculated via direct Limma-integration for analytical stability",
x = "log2 Fold Change (Disease vs Control)",
y = "-log10 p-value",
color = "Statistically Significant (p < 0.05)")
# 6. Functional Interpretation (Roadmap Step 5)
# In practice, df_results can be passed to LION-web or lsea() for pathway enrich
```

Supplementary Table

Table S1: R Package Selection Strategy and Technical Documentation

R Package	Source	Version*	Last Update	Intended Analytical Use	Refs
xcms	Bioconductor	3.22.0	2024-05	Peak picking, RT correction, and feature grouping.	[10,43]
MSnbase	Bioconductor	2.26.0	2024-05	Infrastructure for MS data handling and metadata.	[11]
LipidMS 3.0	CRAN	3.0.2	2023-11	Rule-based lipid identification and annotation.	[12]
lipidr	Bioconductor	2.14.1	2024-04	QC filtering, PQN normalization, and data mining.	[13]
impute	Bioconductor	1.74.1	2024-04	kNN-based imputation for missing values (MAR).	[25]
limma	Bioconductor	3.56.2	2024-05	Linear modeling and differential abundance testing.	[20]
clusterProfiler	Bioconductor	4.8.2000	2024-05	Functional enrichment and pathway analysis.	[15]
data.table	CRAN	1.17.0	2024-12	High-performance data wrangling and fast I/O.	[18]
vegan	CRAN	2.6-10	2024-12	Ecological diversity metrics and NMDS ordination.	[35]
tidyverse	CRAN	2.0.0	2024-03	Data manipulation and visualization (ggplot2).	[41,42]

*Versions reflect the local environment used for roadmap validation (R 4.3.0 "Already Tomorrow", Bioconductor 3.17).
